# DIANA: A deep learning-based paprika plant disease and pest phenotyping system with disease severity analysis

**DOI:** 10.3389/fpls.2022.983625

**Published:** 2022-10-06

**Authors:** Talha Ilyas, Hyungjun Jin, Muhammad Irfan Siddique, Sang Jun Lee, Hyongsuk Kim, Leon Chua

**Affiliations:** ^1^ Core Research Institute of Intelligent Robots, Jeonbuk National University, Jeonju-si, South Korea; ^2^ Division of Electronic and Information Engineering, Jeonbuk National University, Jeonju-si, South Korea; ^3^ Department of Plant Science and Plant Genomics and Breeding Institute, Seoul National University, Seoul, South Korea; ^4^ Department of Horticultural Science, North Carolina State University, Mountain Horticultural Crops Research and Extension Center, Mills River, United States; ^5^ Department of Electrical Engineering and Computer Sciences, University of California at Berkeley, Berkeley, CA, United States

**Keywords:** plant phenotyping, disease severity analysis, diseases and pests recognition, detection, deep learning

## Abstract

The emergence of deep neural networks has allowed the development of fully automated and efficient diagnostic systems for plant disease and pest phenotyping. Although previous approaches have proven to be promising, they are limited, especially in real-life scenarios, to properly diagnose and characterize the problem. In this work, we propose a framework which besides recognizing and localizing various plant abnormalities also informs the user about the severity of the diseases infecting the plant. By taking a single image as input, our algorithm is able to generate detailed descriptive phrases (user-defined) that display the location, severity stage, and visual attributes of all the abnormalities that are present in the image. Our framework is composed of three main components. One of them is a detector that accurately and efficiently recognizes and localizes the abnormalities in plants by extracting region-based anomaly features using a deep neural network-based feature extractor. The second one is an encoder–decoder network that performs pixel-level analysis to generate abnormality-specific severity levels. Lastly is an integration unit which aggregates the information of these units and assigns unique IDs to all the detected anomaly instances, thus generating descriptive sentences describing the location, severity, and class of anomalies infecting plants. We discuss two possible ways of utilizing the abovementioned units in a single framework. We evaluate and analyze the efficacy of both approaches on newly constructed diverse paprika disease and pest recognition datasets, comprising six anomaly categories along with 11 different severity levels. Our algorithm achieves mean average precision of 91.7% for the abnormality detection task and a mean panoptic quality score of 70.78% for severity level prediction. Our algorithm provides a practical and cost-efficient solution to farmers that facilitates proper handling of crops.

## 1 Introduction

In the agricultural sector, plant diseases are responsible for major economic losses worldwide, affecting a country’s revenue and the livelihood of its people (Savary et al., 2012). They link directly to the sustainable food production and safety. Accurate, precise, and reliable quantification of disease severity and intensity is one of the main challenges in plant phytopathology (Pethybridge and Nelson, 2015; Donatelli et al., 2017). A reliable and precise assessment of plant diseases and their intensity (severity) can help in pesticidal management, disease forecast, crop loss modeling, and spatiotemporal modeling of epidemics (Gaunt, 1995; Kranz and Rotem, 2012). There exist three different techniques for plant disease recognition, i.e., chemical, manual, and optical. Chemical techniques involve the use of various chemicals and analysis of their reaction to a particular pathogen to identify diseases (Alvarez, 2004; Chaerani and Voorrips, 2006; Gutiérrez-Aguirre et al., 2009). The second method for plant disease inspection is related to manual labor (Bock et al., 2010; Martinelli et al., 2015). In these methods, detection is done by field experts *via* visual (manual) analysis of abnormal plant regions. Due to advances in machine learning and computer vision, the plant disease phenotyping trend is shifting toward optical identification. Digital optical phenotyping consists of two steps. The first step involves disease-specific feature extraction (from digital images) *via* either hand-crafted feature-based (Lowe, 2004; Dalal and Triggs, 2005) methods or deep learning-based methods (Krizhevsky et al., 2012; Szegedy et al., 2017; Tan and Le, 2019). The second step involves classification of the disease based on the extracted features. The application of deep learning methods has risen considerably with the advent of media and technology. Along with it, the need for fast and accurate approaches is rising for better and more reliable results.

Each methodology for plant disease phenotyping has its own potential pitfalls. For instance, manual plant assessment is time consuming, labor extensive, and prone to human error and uncertainty. Being a human activity, the precision and accuracy of the analysis drops due to fatigue and the physically (and mentally) tiring nature of the assessment task (Nutter Jr et al., 1991). Moreover, a single (intra) field expert or a team (inter) of experts while assessing plant diseases can provide completely different assessments of the same sample depending upon their visual analysis (Nutter Jr et al., 1993). This introduces a large variability in inter and intra rater (field expert) assessment of different plant diseases which reduces the overall reliability diagnosis. Chemical-based methods can be regarded as destructive methods for analyzing plant diseases, because they entail a chemical analysis of the diseased region of the plant or removal of the infected leaf from the plant, and tests on it are performed in an isolated environment inside the laboratory (Alvarez, 2004; Chaerani and Voorrips, 2006; Gutiérrez-Aguirre et al., 2009).

The process of optical disease phenotyping is a non-destructive method that involves the use of digital plant images. These images are used to extract features that are then used to classify the type of disease infecting the plant. Feature extraction is performed by either deep learning-based methods or hand-crafted feature-based methods. In hand-crafted feature-based methods, the performance is highly dependent upon the features implied during the development of the algorithm by a field expert (Dalal and Triggs, 2005; Nanni et al., 2017), whereas in the case of deep learning-based feature extractors, more robust and adaptive features can be learned on the fly, making these methods superior to the hand-crafted-based feature methods. Furthermore, plant diseases can have different visual traits even within the same class, due to different pathogens attacking at several locations and causing different stage infections. This results in several interclass and intra-class variations in the appearance of the different diseases, making the optical plant disease phenotyping task even more challenging.

Recent studies in plant disease phenotyping have shown considerable progress. The accuracy of these frameworks depends heavily on the extraction and selection of apparent disease features. We can divide the recent works into three categories: (a) image-based disease classification, (b) pixel-level classification of abnormalities in plants, and (c) region-based classification and localization of plant anomalies. The first method predicts if an image contains an object of interest or object class (i.e., what), the second method entails a pixel-wise classification of plant image into healthy and non-healthy regions (i.e., what and where), and the third gives information on the classes and locations of all abnormal instances (i.e., what and where) present in the image. These approaches have limited capacities in terms of delivering an accurate assessment of disease symptoms in plants.

In this work, we take a step further toward deep plant phenotyping tools by proposing a system that can generate user-friendly specific descriptive phrases, which describes the location (where), severity (intensity), and type (what) of all abnormalities present in the plant. The main contributions of this paper can be summarized as follows.

1. For the identification and evaluation of the severity of paprika plant diseases, a powerful end-to-end trainable deep learning system is proposed.
2. To notify the farmer of the plant’s present health status, our proposed algorithm produces user-friendly phrases.
3. A new dataset for diagnosing paprika plant disease is introduced. Our dataset includes disease-specific labels of six different paprika plant diseases, along with their 11 severity stages of infection, in contrast to existing datasets in literature which only provide disease-specific labels. A comparison of the proposed dataset’s features with existing plant disease databases is shown in **Table 1**.
4. To the best of our knowledge, we are the first ones to analyze various severity stages of plant diseases *via* deep learning-based methods.

**Table 1:** Summary of a few datasets available in literature for plant disease recognition.

Author (year)	Plant	Background	Environment	Disease categories	Severity stages	Framework	Backbone
Fuentes et al. (2017)	Tomato	Complex	Outdoor	9	–	Faster-RCNN	VGG-16
Kundu et al. (2021)	Peral Millet	Complex	Outdoor	2	–	CNN	Inception-ResNet
Liu and Wang (2020)	Tomato	Complex	Outdoor	12	–	YOLO	DarkNet-53
Amara et al. (2017)	Banana	Simple	–	3		CNN	LeNet
Kawasaki et al. (2015)	Cucumber	Simple	–	3	–	CNN	Custom
Zhong and Zhao (2020)	Apple	Simple	Indoor	6	–	CNN	DenseNet-121
Nie et al. (2019)	Strawberry	Simple	Outdoor	4		Faster-RCNN	ResNet-50
**Proposed**	Paprika	Complex	Outdoor	6	11	DIANA	EfficientNet-B3

**Figure 1:**
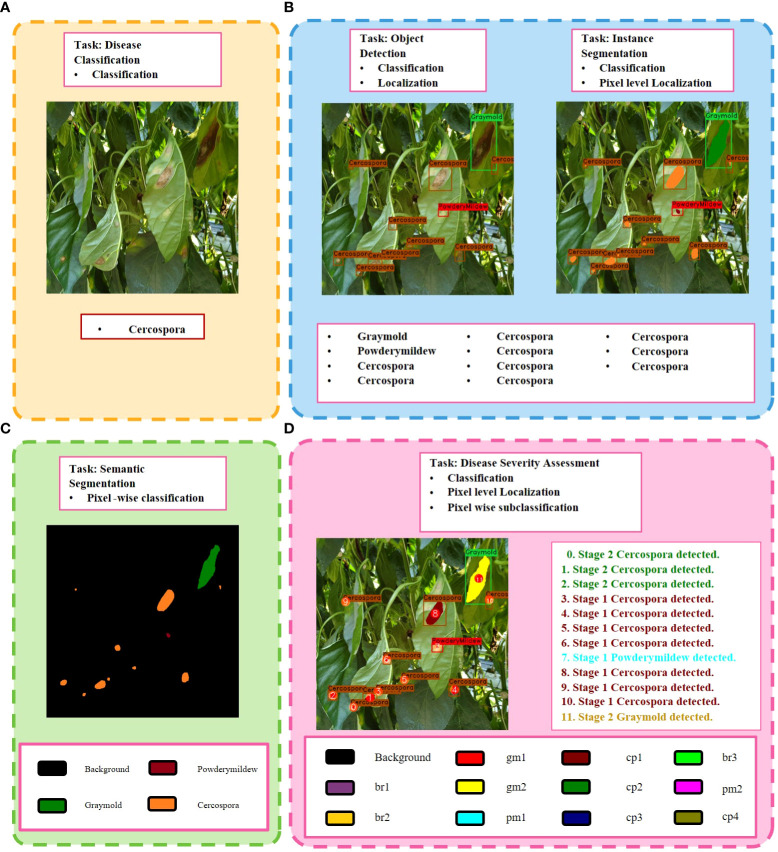
Key differences between different frameworks of plant disease phenotyping. **(A)** Image-based disease classification. **(B)** Pixel-level classification of plant abnormalities. **(C)** Region-based classification and localization of plant anomalies. **(D)** Our proposed Disease Severity Analysis task, which provides more detailed information regarding all anomalies detected in the plant. The color pallet in **(C, D)** shows the colors given to a severity level of a specific disease throughout this paper. Best viewed in color.

**Figure 1** gives a graphical abstract on the key difference between recent approaches and our proposed approach. To achieve our goal, we propose a hybrid deep learning-based disease analyzer (DIANA), which combines two state-of-the-art CNNs in one framework to generate global descriptive sentences. Our proposed approach consists of three main units:

- The disease detector and localizer (DDL) unit, which should localize and classify different types of abnormalities (disease and pest damage) present in the plant precisely and efficiently.
- The disease severity analyzer (DSA) unit, which should recognize the intensity of the diseases present in the plant by analyzing local regions provided by the DDL.
- Information from both these units combined by an integration unit (IU) to generate specific sentences which provide a full description of anomalies present in the plant. Each sentence contains information regarding the intensity and type of abnormality detected at a particular location on plant. **Figure 1D** depicts the goal of our network.

Finally, our framework generates a set of fine-segmented regions, bounding boxes, and specific phrases that describe the type and intensity of the damage present in plant. Considering how the aforementioned three units can be combined to deliver results, there are two possible strategies. In this article, we go over both techniques. See *System overview* for details.

## 2 Related work

Plant disease detection is a critical and important topic which has been explored throughout the years in context of deep learning-based plant phenotyping. It is driven by the need to produce high-quality, nutritious food and to reduce economic losses. However, cost-effectiveness, usability, accuracy, and reliability are some desirable characteristics that must be taken into account when developing such systems (Gehring and Tu, 2011; Dhaka et al., 2021; Jagtiani, 2021). Recent deep learning studies have addressed the automated diagnosis of plant diseases in several plant species in non-destructive ways. The approaches can be mainly classified into two types: (a) image-based disease recognition (classification) and (b) region-based disease recognition (localization and classification).

### 2.1 Image-based disease recognition

In the case of image-based disease detection, features pertaining to a specific disease are extracted from an image using CNN, and based upon these features, the detected disease is assigned a class. Some recent applications include recognition of different diseases in several crops, like banana (Amara et al., 2017), apple (Liu et al., 2017), tomato (Fuentes et al., 2018; Liu and Wang, 2020), cucumber (Kawasaki et al., 2015), strawberry (Nie et al., 2019), and pearl millet (Kundu et al., 2021). Deep learning-based models can serve as powerful feature extractors for various computer vision tasks. Recently, deep learning techniques have dominated the PlantCLEF challenge (Goëau et al., 2014). Choi (2015) used Inception Net (Szegedy et al., 2015) to classify 1,000 plant species in the PlantCLEF challenge and won the PlantCLEF 2015 challenge. Ghazi et al. (2017) combined the two state-of-the art deep neural networks (NNs) and achieved even higher validation accuracy. Mohanty et al. (2016) classified 26 different types of diseases in 14 crop species using Inception Net (Szegedy et al., 2015) and AlexNet (Krizhevsky et al., 2012). They also showed that particular diseases in a crop can be efficiently detected using transfer learning (Huang et al., 2017b). However, one major disadvantage of this work was that its analysis was based solely on in-lab (inside laboratory) images, not under real field conditions. Consequently, their experiments did not cover all the variables. Ferentinos (2018) performed an interesting experiment while classifying 58 different plant diseases of 25 distinct plants. They collected two separate sets of images, one containing in-lab images and the other containing field images (images collected in real-field scenarios). Their experiments showed that while using in-lab images, the system was able to achieve a high accuracy of 99.53%. However, the test accuracy dropped considerably while using field images. This experiment showed that the disease classification is significantly harder and more complex under real field situations.

### 2.2 Region-based disease recognition

On the other hand, region-based disease detectors can detect multiple instances of distinct or the same disease in an image, based upon the features obtained from different regions of the image. It is also worth mentioning that, even though aforementioned techniques serve as valuable tools for feature extraction and classifying different diseases present in images, their performance is quite limited under real-field conditions (Ferentinos, 2018; Hussain et al., 2021). These methods do not cover all the variables constituting real-world scenarios like disease severity, presence of multiple abnormalities in the same sample, complex background, and nearby objects. In contrast, some recent region-based disease detectors have shown good performance in real-field settings. Arsenovic et al. (2019) collected 79,265 images and constructed a large-scale plant disease dataset. They proposed a two-stage plant disease net (PD-Net) which further consists of two subnetworks PD-Net1 and PD-Net2. PD-Net1 uses the YOLO (Redmon et al., 2016) algorithm to detect plant leaves and PD-Net 2 to classify the leaves into different categories. Under real-field conditions, their method was able to achieve a 91.6% mean average precision (mAP) for the detection task and 93.67% accuracy for the classification task. Jiang et al. (2019) proposed a real-time system for apple plant disease and pest recognition. Szegedy et al. (2015) and Szegedy et al. (2017) incorporated Inception-Module, and Liu et al. (2016) incorporated rainbow concatenation with a single-stage object detector (SSD); they were able to achieve a 78.8% mAP, with a detection speed of 23.13 frames per second (FPS). They constructed their apple leaf disease dataset using both in-lab and real-field images, thus considering all the variables of real-field scenario. Nie et al. (2019) proposed a unique method for detecting strawberry verticillium wilt. Instead of directly classifying the whole plant as having verticillium wilt or not, they first classified and detected young petioles and leaves in the image and then used the detected components to decide whether the whole plant is infected or not. They further improved their accuracy by adding an attention mechanism in Faster-RCNN’s (Ren et al., 2015) backbone and was able to achieve a mAP of 77.54%. Tian et al. (2019) made substantial changes in the YOLO-v3 (Redmon and Farhadi, 2018) architecture to detect anthracnose damage in apple plants. They were able to achieve a 95.57% mAP by changing the backbone of YOLO-v3 with Dense-Net (Huang et al., 2017a) and also optimizing feature extraction layer of YOLO-v3.

Fuentes et al. (2017) proposed three different architectures, i.e., Faster-RCNN, SSD, and R-FCN (Dai et al., 2016), for tomato plant disease and pest localization and classification and compared their performance before and after applying different data augmentation techniques. The best results of 83.06% mAP were obtained by Faster-RCNN having a VGG-16 backbone. Their experiments showed that having a good backbone and using appropriate data augmentation techniques can boost the network’s performance by more than 25% (mAP). Fuentes et al. (2018) further improved their results to 96% on the tomato anomaly detection task while using the same architecture (i.e., Faster-RCNN with VGG-16 backbone). For improving their results, they proposed a simple yet effective strategy by the name of Refinement Filter Bank whose task was to remove false positives from the network’s predictions. The Refinement Filter Bank consisted of independently trained CNNs (one for each class); each CNN was tasked with removing misclassified samples from its corresponding class. Fuentes et al. (2019) developed a framework that is capable of not only efficiently detecting plant anomalies (92.21% mAP) but also describing the location and class of the disease in a human-readable format. Their framework consisted of a combination of Faster-RCNN (for detecting anomalies) and multiple long–short-term memory (LSTM) units. Liu and Wang (2020) proposed an improved YOLO v3 architecture for detecting 12 different diseases in tomato plants. They were able to achieve a 96.91% mAP even without the use of the Refinement Filter Bank (Fuentes et al., 2018) while retaining a processing speed of 49 FPS.

### 2.3 Plant disease severity assessment

Although the aforementioned works showed remarkable performance in plant disease and pest detection task, none of them focused on analyzing the intensity of the detected infection in plants, whereas a reliable and accurate evaluation of plant disease intensity can aid in pesticidal control, disease forecasting, spatiotemporal epidemic modeling, yield-loss prediction, crop damage management, etc. Despite the importance of the disease severity analysis (DSA) task, little research has been conducted in this field. Wang et al. (2017) separated apple black rot images from the PlantVillage (Hughes and Salathé, 2015) dataset. They further subclassified these images into four classes depending upon the severity of black rot disease with the help of field experts. The classification accuracy of Inception Net on the whole PlantVillage dataset is about 98.24% (Mohanty et al., 2016). In contrast to the DSA task, even after fine-tuning Wang et al. were only able to achieve 83% accuracy on this newly annotated dataset. Their experiments showed fine-grained DSA task is substantially more difficult than simple classification of different diseases since in this case there exists a high intra-class similarity and low interclass variance (Xie et al., 2015). Moreover, Pukkela and Borra (2018) summarized the findings of various machine-learning-based plant DSA systems. They also outlined popular metrics used in classical DSA tasks to measure the performance of an algorithm, e.g., ratio of infected area (RIA), lesion color index (LCI), damage severity index (DSI), and infection per region (IPR). Shrivastava et al. (2015) proposed a system which performs segmentation *via* classical color thresholding and then performed morphological filtering operations to detect foliar diseases in soybean plants. Mahlein et al. (2012) proposed the use of hyperspectral imaging for the DSA task along with other image processing algorithms. Li et al. (2013) employed image thresholding techniques to obtain the infected tissue area of the leaf and then calculated the RIA to estimate the disease severity.

All the DSA strategies listed above do not consider the following common occurrences of real-field scenarios in their analysis:

- The background (BG) of images will be cluttered and complex in real life.
- The infected leaves might be partially occluded by surrounding objects like crop stalks, tree branches, and fruits.
- The effect of different lighting conditions such as weather, and angle differences on the appearance of the infected area of the plant.
- Fuzzy boundaries between healthy and infected parts of the leaf.
- In various phases of development (severity stages), and even in various locations, the same disease can have completely different characteristics.
- Multiple instances of similar or different diseases and pests might appear at the same location at the same time.
- In cases when dead leaves are in close proximity to the infected leaves, it can be quite challenging to differentiate between them.

We take all the abovementioned scenarios into account while developing our framework disease severity analysis (DIANA), for simultaneous disease recognition, localization, and severity analysis. We perform detailed experiments to analyze the performance of our proposed unified network in real-life scenarios both quantitatively and qualitatively. For quantitative analysis, we use three state-of-the-art metrics mean average precisions (mAPs), mean panoptic quality (mPQ), and mean intersection over union (mIOU) to evaluate its robustness. As for qualitative analysis, we show multiple cases where our network successfully detected the location, category, and severity of the disease in complex and cluttered surroundings. Our paprika plant dataset is also collected under real-field conditions, with different lightings and from different farms across Republic of Korea.

## 3 Materials and methods

### 3.1 Dataset construction

The demand for paprika worldwide is over 50,000 tons per year, and the number is continuously increasing. Paprika, tomato, tobacco, and potato are all members of the *Solanaceae* family (Shah et al., 2013). Paprika being susceptible to various diseases, including potato virus Y, cucumber mosaic, and tobacco mosaic viruses, affecting these other plants, makes it crucial. As a result, these crops are not ideal for a 3-year rotation and should not be planted near paprika. However, its demand is increasing; many farmers in Southern Africa are converting from tobacco to paprika, which may have an impact on pricing and production (Aminifard et al., 2010). Due to the aforementioned reasons, we considered paprika crop for the development and evaluation of our disease severity analysis framework (DD-RCNN).

#### 3.1.1 Data acquisition

Paprika images were collected from several farms across Jeonju-si District, Jeollabuk-do, Republic of Korea, during the growing season 2019. The data acquisition was carried out using a 24.1-megapixel Canon EOS 200D-based platform with a CMOS sensor. All the images contain diverse background artifacts as well as other objects found in the field. Moreover, images captured during different time periods, under varying lightings and weather conditions, would help to make data more generalized and representative of real-field scenarios. Overall, our collected dataset has the following characteristics:

- Different resolution and aspect ratio images.
- Samples at several severity (intensity) stages of infection.
- Different infected areas of plants including stem, leaves, and fruits are captured.
- Varying plant sizes.
- Background artifacts of the farmhouse and surrounding objects.

Following the above protocol, we acquired 6,000 raw images. The images were saved in JPEG format at a high resolution, to avoid being limited in available resolution at subsequent processing stages.

#### 3.1.2 Data analysis and distribution

We started with primary data filtering and removed the images which were blurred. After the primary filtering step, we ended up with 5,900 raw images. Out of the total dataset available, we randomly split data into two. One split has 80% data for training and validation, and the second split contains 20% data for testing. The detailed statistical distribution of dataset is given in **Table 2**. Stages 1 to 4 correspond to the severity level of the disease (explained shortly).

**Table 2:** Statistical distribution of the paprika plant disease dataset.

Class	Annotated instances	Proportion (%)	Class	Annotated instances	Proportion (%)
Blossom end rot	7,631	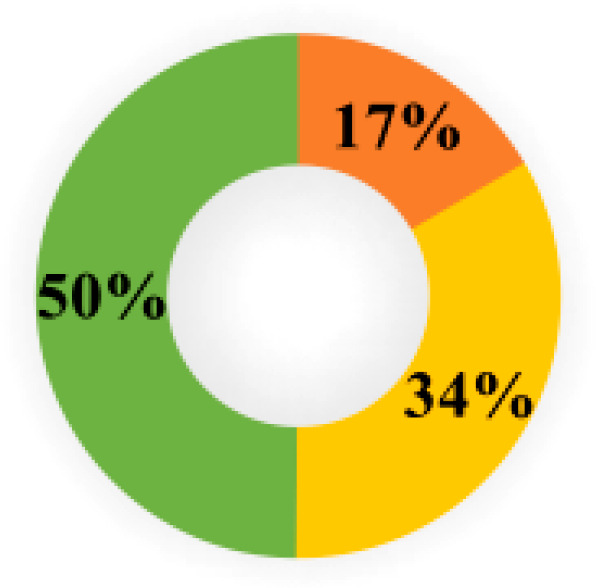	Powdery mildew	7,635	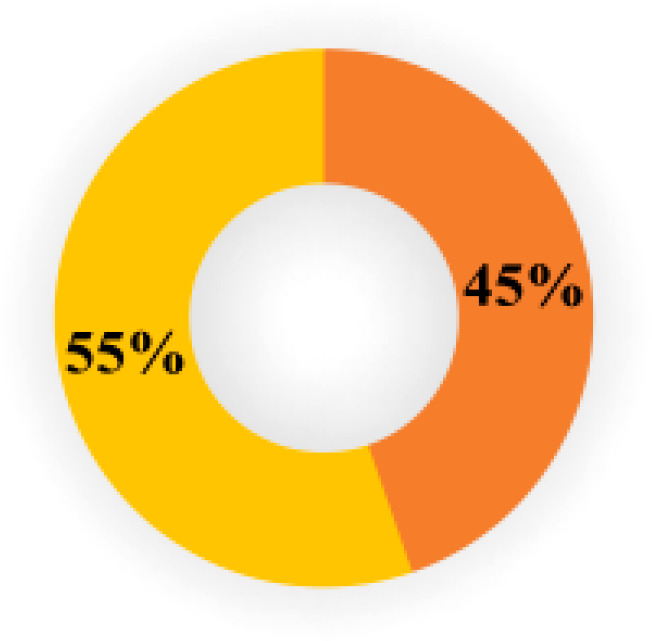
Gray mold	6,046	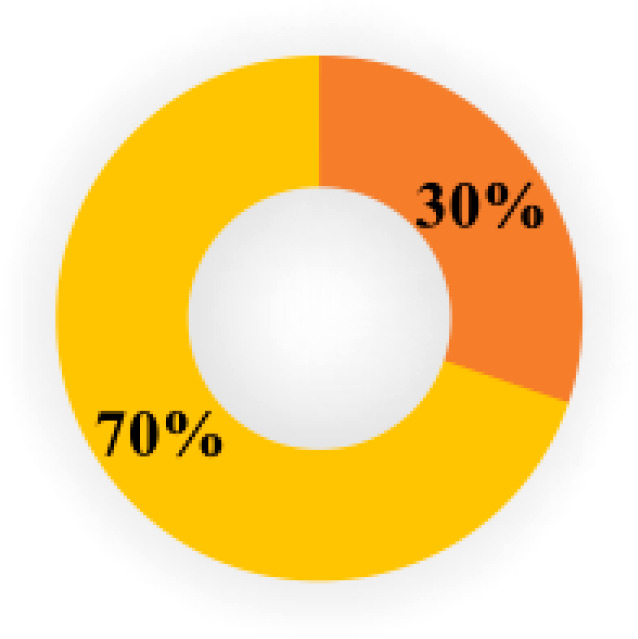	Cercospora	14,238	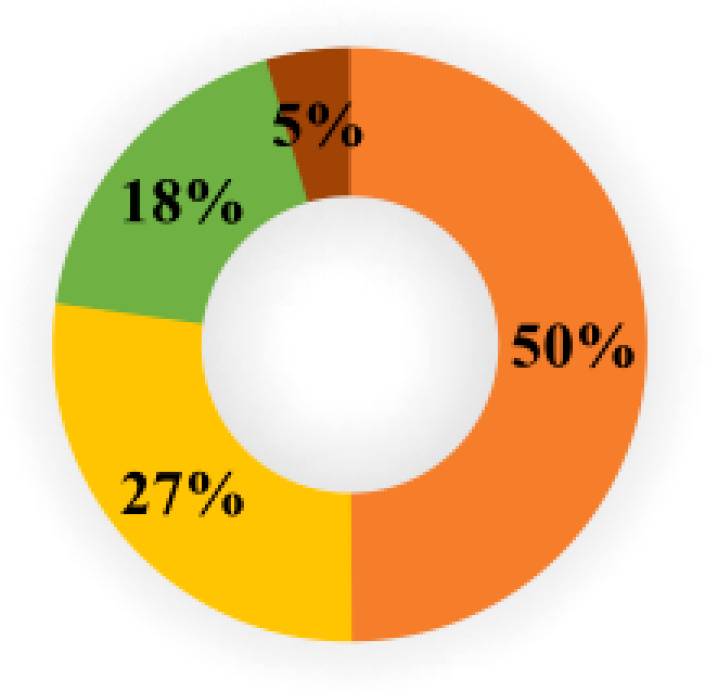
Snails and slugs	8,668	–	Spider mite	5,677	–

◼ Stage 1 ◼ Stage 2 ◼ Stage 3 ◼ Stage 4

**Figure 2:**
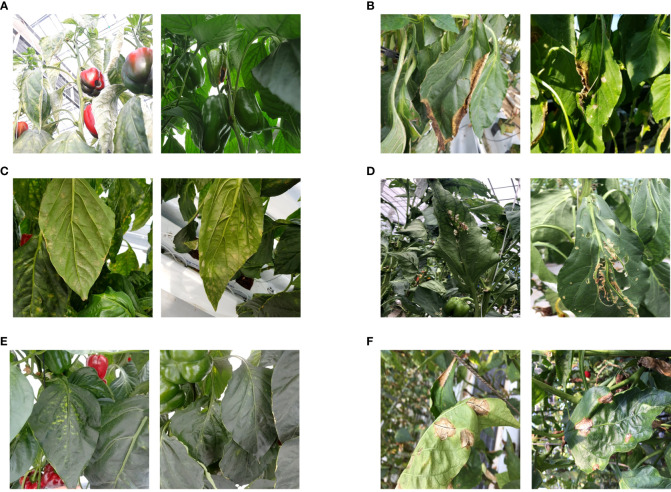
Representative instances of each class **(A)** blossom end rot, **(B)** gray mold, **(C)** powdery mildew, **(D)** snails and slugs, **(E)** spider mite, and **(F)** *Cercospora*.

Then, with the help of field experts, we analyzed the data and were able to find six abnormalities in the collected dataset of paprika. **Figure 2** shows the anomalies present in the paprika dataset. Among six abnormalities, four were bacterial of fungal diseases like (a) blossom end rot, (b) powdery mildew, (c) gray mold, and (d) *Cercospora* leaf spots (henceforth referred to as *Cercospora* for simplicity). The remaining two abnormalities were caused by insects and pests. We labeled them as follows: (a) snails and slugs and (b) spider mite. Some representative instances belonging to each class are shown in **Figure 2**.

In the second stage of data processing, we further subclassified the viral and fungal diseases, with the help of field experts, depending on the intensity of the damage visible on the plant leaves or fruits. We subclassified the data into a maximum of four stages of severity (from 1 to 4). While subclassifying the disease according to their severity, the following pointers were considered.

##### 3.1.2.1 Blossom end rot

This disorder’s initial symptom is a small, water-soaked discoloration on the fruit’s blossom end (stage 1). The lesions typically become sunken into the fruit as they grow larger, turning leathery and dark brown or black (stage 2). Bacteria and fungus may infiltrate the lesion over time, causing a soft, watery rot (stage 3) (Neave, 2018; Hansen, 2020).

##### 3.1.2.2 Powdery mildew

Powdery mildew is a fungal disease; when paprika gets infected by it, fluffy colonies appear on the top surface of the leaf as the first sign. Because the fungus infects the plant through the stomata, and there are more stomata on the underside of the leaf, the fluffy colonies appear predominantly on the underside of the leaf in pepper. Yellow dots may be seen on the upper side (stage 1). The fluffy colonies emerge on the upper surface of the leaves when the intensity increases (stage 2) (Koppert).

##### 3.1.2.3 Gray mold

The symptoms start as a light-brown, water-soaked, slimy lesion on wounded fruit, petals, or senescing leaves (stage 1), and slowly the afflicted areas turn dark-brownish-gray and powdery-looking as spores form (stage2) (Schwartz et al., 2005).

##### 3.1.2.4 Cercospora

The leaves exhibit small, circular, or irregular dark-brown spots, with or without white centers (stage 1). As the spots enlarge in size, the center becomes light brown surrounded by dark-brown rings (stage 2). After that, spots coalesce to form large irregular patterns (stage 3). In extreme cases, the coalescing spots create a hole in the leaf (Stage 4) (Kohler et al., 1997).

**Figure 3:**
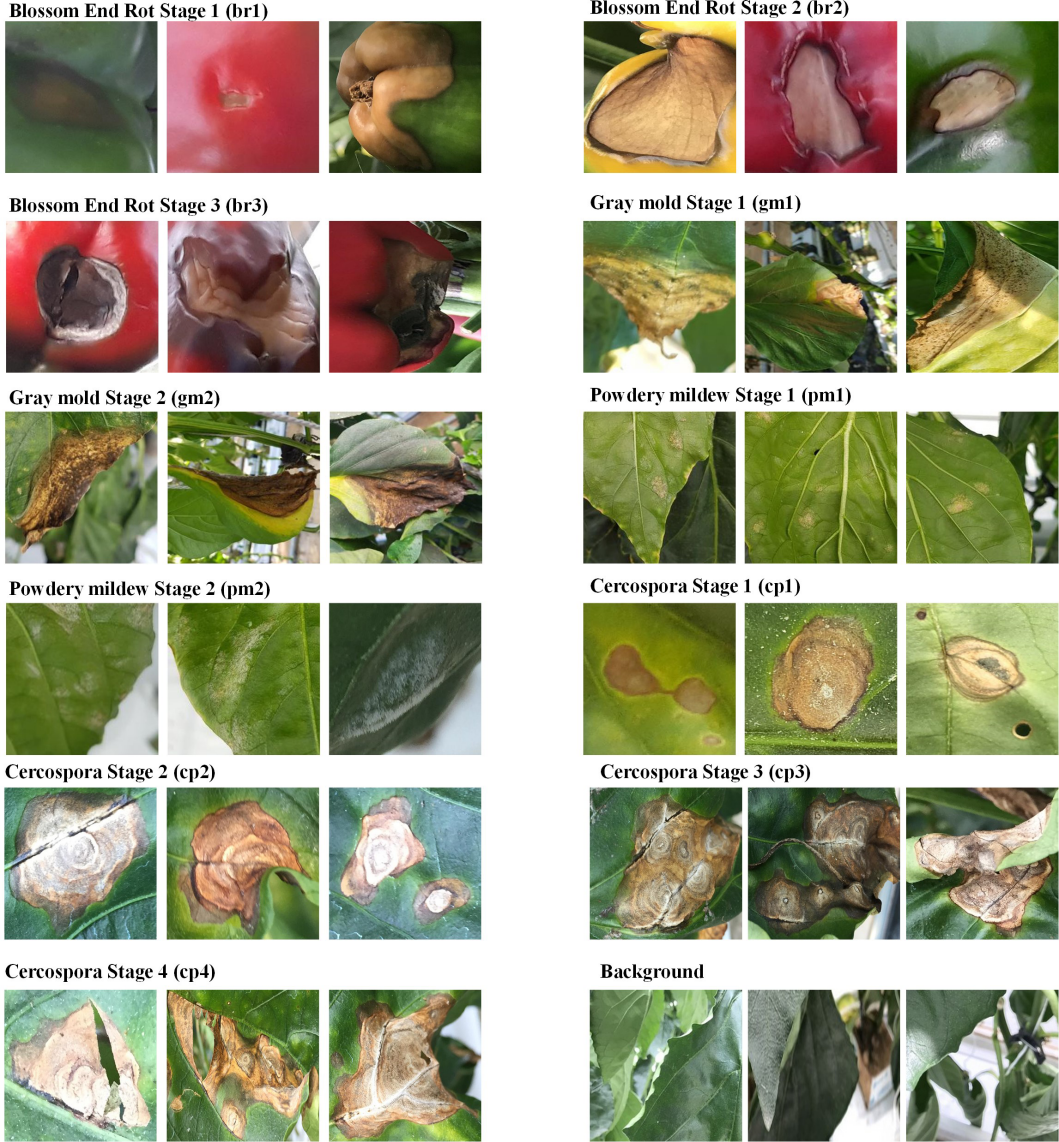
Image showing visual differences of various severity stages belonging to the diseases present in the paprika dataset.

Some typical cases belonging to each severity stage are shown in **Figure 3**. To improve the performance of the proposed framework, we employ various custom data augmentation techniques for training our network, details of which can be found in the supplementary materials provided.

## 4 System overview

In this work, we take a step further toward deep plant disease phenotyping tools by proposing a system that can generate user-friendly and meaningful descriptive sentences which describe the location (where), severity (intensity), and type (what) of all abnormalities present in the plant. Our main goal is to locate and recognize the intensity (severity) level of different plant diseases, specifically for our newly constructed paprika plant dataset. Considering how the aforementioned three units, i.e., DDL, DSA, and IU, can be combined to deliver results, there are two possible strategies. In this article, we go over both techniques.

### 4.1 Proposed approach

**Figure 4:**
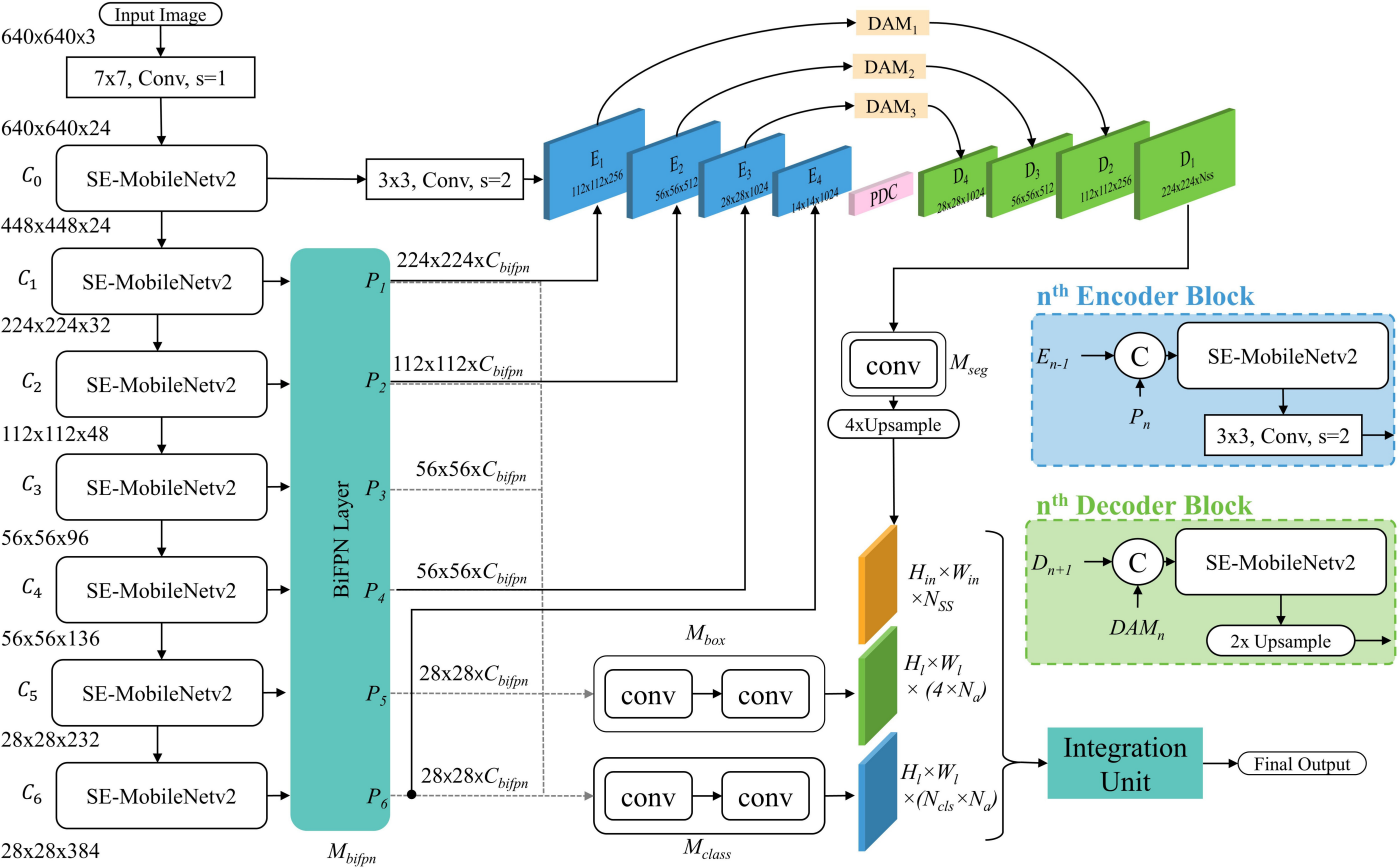
Overall architecture of the DIANA framework. The whole framework is end-to-end trainable.

Our proposed approach combines the DDL and DSA units in a single framework such that both can be trained in an end-to-end fashion and their learnings complement one another, producing a better and more consistent performance. The overall diagram of the proposed framework is shown in **Figure 4**.

In the following subsections, first, we describe the disease detector and localizer (DDL) unit and then explain the construction of the disease severity analyzer (DSA) unit. Finally, we describe how the integration unit works to integrate predictions and generate meaningful phrases.

#### 4.1.1 Disease detector and localizer unit

The goal of the DDL unit is to detect and recognize the type and location of disease and pest candidates in the image. To detect our target, we must first properly locate the box in which it is contained, as well as determine the class to which it belongs. The proposed DDL unit consists of a CNN backbone to extract robust multiscale features and a feature network for cross-scale feature fusion for generating reliable and precise predictions, constituting an object detector (OD) pipeline. These cross-scale weighted features are also shared with the DSA unit (explained in next section) for joint optimization and robust predictions.

For extracting robust multiscale features, we utilize EfficientNet-B3 as the backbone of our DDL unit. The width (number of channels), depth (number of layers), and resolution (input resolution) of the network are calculated using the compound scaling method (Tan et al., 2020). Each block of EfficentNet-B3 consists of inverted residual blocks (Sandler et al., 2018) further enhanced by squeeze-excitation modules (Hu et al., 2018). We refer to these modules as SE-MobileNetv2 throughout this paper. The complete architecture of EfficentNet-B3 is shown in **Figure 4**. From EfficientNet-B3, feature maps are extracted at seven different levels, namely, {C_0,_ C_1, …,_ C_6_}; here the subscript represents the convolutional block from which the feature maps are coming.


(1) $$C_{b=\left\{1,2,\ldots ,7\right\}}=\;N\left(X_{H_{in},W_{in},C_{in}}\right)$$

Here *N* represents the EfficnetNet-B3 network, X is the input tensor having dimensions H_in_,*W_in_*, *C_in_* and C_b_ is the output of the bth block of CNN, such that C_b_ ∈ℝ*^H^_b_^,W^_b_^,C^_b_ H_b_*, *W_b_*, *C_b_* being the dimensions of features output by bth block. These multiscale feature maps output by EfficnetNet-B3 are first passed through a single 3 × 3, stride=1 conv layer (i.e., f_1_^3×3^ to limit the number of channels to a constant value of C_bifpn_.


(2) $${\hat{C}}_{b=\left\{1,2,\ldots ,6\right\}}=\;f_{1}^{3\times 3}\left(C_{b=\left\{1,2,\ldots ,6\right\}}\right)$$

Where Ĉ_b={1,2,…6}_ ∈ℝ^〈H^_b_^,W^_b_^,C^_bifpn_^〉^. After channel reduction, these multiscaled features are sent to the bREGt al., 2020) for weighted feature fusion. As different-level features contribute unequally to the output feature map (Ilyas et al., 2022), therefore, at each level BiFPN recalibrates the features according to their importance and fuse them together. Each BiFPN outputs P_l_-recalibrated feature maps, where l is the number of the pyramid level.


(3) $$P_{l=\left\{1,2,\ldots ,6\right\}}=\;BiFPN\left({\hat{C}}_{b\langle H_{b},W_{b},C_{bifpn\rangle }}\right)$$

where *P*_*l*_∈*ℝ* ^*H*_*l*_,*W*_*l*_,*C*_*bifpn*_^ and H_l_, *W_l_*, *C_bifpn_* are the dimensions of the feature maps output by the lth-level pyramid. As BiFPN do not change the spatial resolution of the input feature maps at any level, in our network, l = b or all pyramid levels. In the proposed architecture, the BiFPN layer is repeated M_bifpn_ times.

Each BiFPN level has two fully convolutional subnetworks (FCNs) linked to it, which utilize these recalibrated feature maps (P_l_) to generate class predictions and to regress from anchor boxes to ground-truth boxes. Each subnetwork shares its parameters across all pyramid levels.

##### 4.1.1.1 Class prediction subnetwork

It applies M_class_ 3 × 3 conv layers to the input feature map of pyramid level l, having a constant width (i.e., number of channels) of C_bifpn_. Each convolutional layer is followed by a ReLu activation, except the last 3 × 3 conv layer which predicts the probability of plant anomaly occurrence for each of the N_a_ anchors and N_cls_ disease categories at each spatial location by applying sigmoid activation at its output (see **Figure 4**).

##### 4.1.1.2 Box regression subnetwork

This network regresses the offset from each anchor box to a nearby ground-truth plant anomaly box. This network is identical to the classification subnetwork except the final layer which predicts four outputs for each anchor (N_a_) at each spatial location (see **Figure 4**). These four outputs (i.e., {x,y,w,h}) predict the relative offset between the anchor and the ground-truth box for each of N_a_ anchor per spatial location.

The output of both these subnetworks is passed onto the integration unit to generate final results.

#### 4.1.2 Disease severity analyzer unit

The purpose of the DSA unit is to recognize the intensity of all the diseases present in the plant. For this task, we fully integrate an encoder–decoder architecture with the DDL unit, which probes the recalibrated multiscale feature maps output by the last BiFPN layer at each corresponding encoder stage, for severity-level prediction (see **Figure 4**).

The proposed DSA unit is built upon on insights from state-of-the-art (SOAT) encoder–decoder architectures (Chen et al., 2017; Peng et al., 2017; Ilyas et al., 2021). The encoder is made up of four encoding stages. Each stage consists of a SE-MobileNet-v2 block followed by a single 3 × 3, stride=2 conv layer, to process and reduce the spatial dimensions of incoming features. In addition to the output of the previous encoder block, the next block also takes input from the corresponding same-resolution BiFPN layer (*P_l_*). In each block, the output is calculated as follows.


(4) $$E_{n}=\;f_{2}^{3\times 3}\left[ℱ\left(E_{n-1}©P_{n}\right)\right],\;n\in \left\{2,3,4\right\}$$

where *ℱ* represents SE-MobileNetv2 and © represents the concatenation operation. Here, E_1_ is calculated from the 0th convolutional block of EfficnetNet-B3 as


(5) $$E_{1\frac{H_{0}}{2},\frac{,W_{0}}{2},C_{0}}=\;f_{2}^{3\times 3}\left[C_{0H_{0},W_{0},C_{0}}\right]$$

Where, H_0_, W_0_, C_0_ represents the spatial dimensions of the 0th convolution block of EfficenNet-B3. At the end of the encoder, the feature maps are processed through a parallel dilation convolution (PDC) module (Ilyas et al., 2021), which probes feature maps at various dilation rates for aggregating global and local contexts.

To modulate the information flow between encoder and decoder, we incorporate the dense attention modules (DAM) (Ilyas et al., 2021) on skip connections between them. The decoder of the DSA unit also consists of four stages. An SE-MobileNetv2 module and a bilinear upsampling operation make up each decoder block. Every decoder block D*_n∈_*_{3,2,1}_ takes two sets of feature maps as an input, one from the output of the previous layer and the other from the corresponding attention module (i.e., DAM). Inside each decoder block, the output is calculated as follows.


(6) $$D_{n}=\overset{⌢}{U}\left[ℱ\left(D_{n+1}©DAM_{n}\right)\right],\;n\in \left\{3,2,1\right\}$$

where $\overset{⌢}{U}$ represents bilinear upsampling operation and D_4_ is simply the output PDC module. The output of the final decoder block is processed through M_seg_ 3 × 3 conv layers and is then upsampled four times to generate the final segmentation maps having dimensions H_in_, *W_in_*, *N_ss_* where N_ss_ is the number of severity stages present in the dataset. These segmentation maps are then passed to the integration unit for further processing.

Both DDL and DSA units are jointly optimized and trained in an end-to-end fashion such that both complement each other and boost the overall performance. Detailed experiments are performed to validate our claims in Results and discussion.

#### 4.1.3 Integration unit

**Figure 5:**
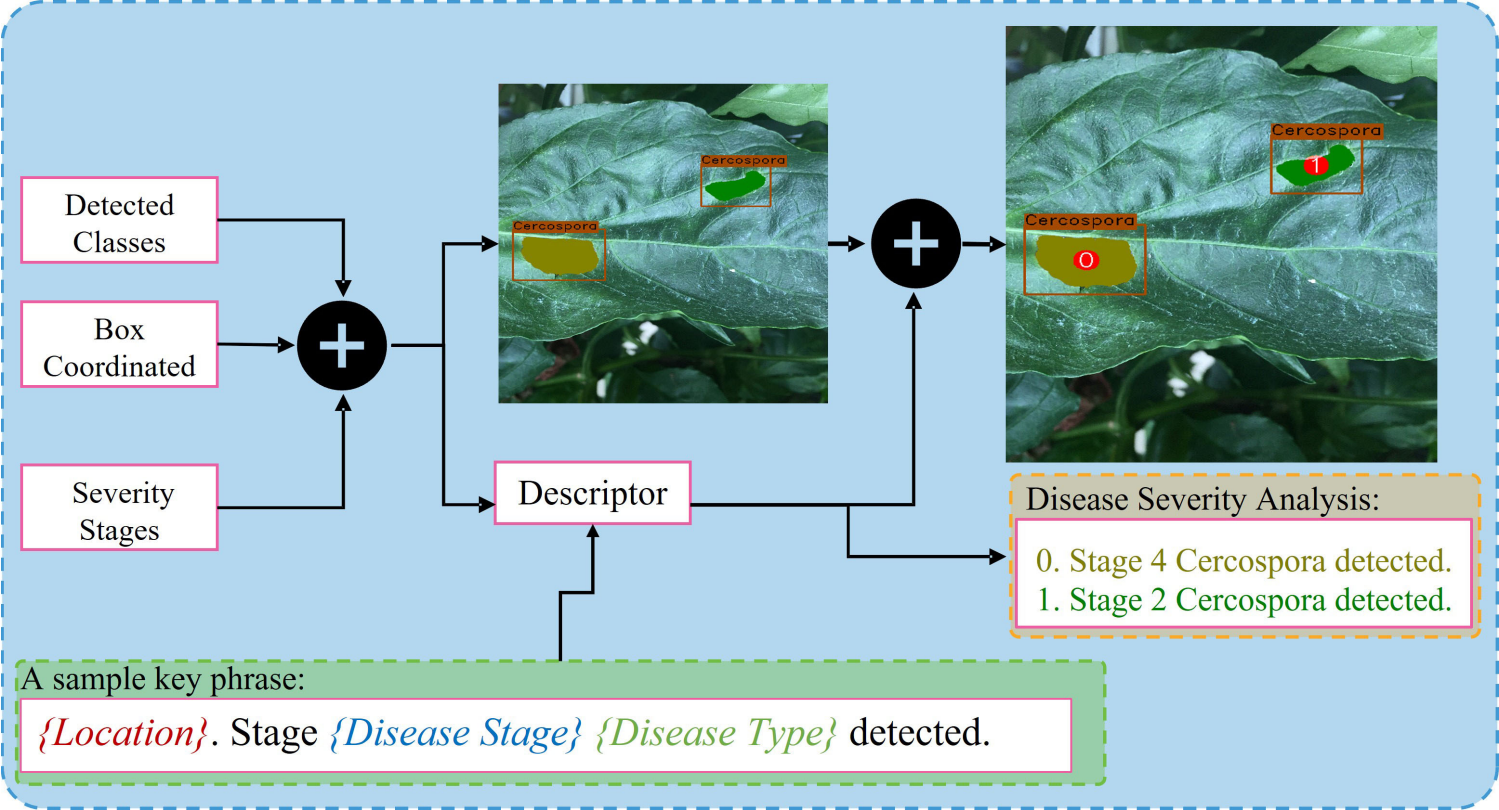
Overview of the process being performed by the integration unit.

The integration unit combines the outputs of the DDL and DSA units. The DDL unit locates and identifies each anomalous instance that exists in a plant. The DSA unit generates severity-specific fine-grained semantic masks, which shows the severity stages of all diseases detected in plant. The integration unit serves three primary purposes: (1) it integrates three distinct forms of information and remaps it onto the original images, (2) it assigns unique IDs to each detected severity stage inside a bounding box, and (3) it creates (user-specified) descriptive words, providing the user with information on the type, location, and intensity of all diseases found in the plant. The whole process is self-contained and automated, allowing the system to produce results quickly. The whole process of the integration unit is summarized in **Figure 5**.

### 4.2 Naïve approach

In this approach, one might consider training a separate object detector (DDL unit) for recognition and localization of plant diseases and then using these discovered disease regions to feed into a classification network (pixel- or region-wise) to determine their severity, i.e., DSA unit. There are two main problems with this type of approach

i. Both networks must be trained independently.
ii. The framework’s two halves cannot communicate with one another. As a result, they cannot enhance one another’s learning or the results.

**Figure 6:**
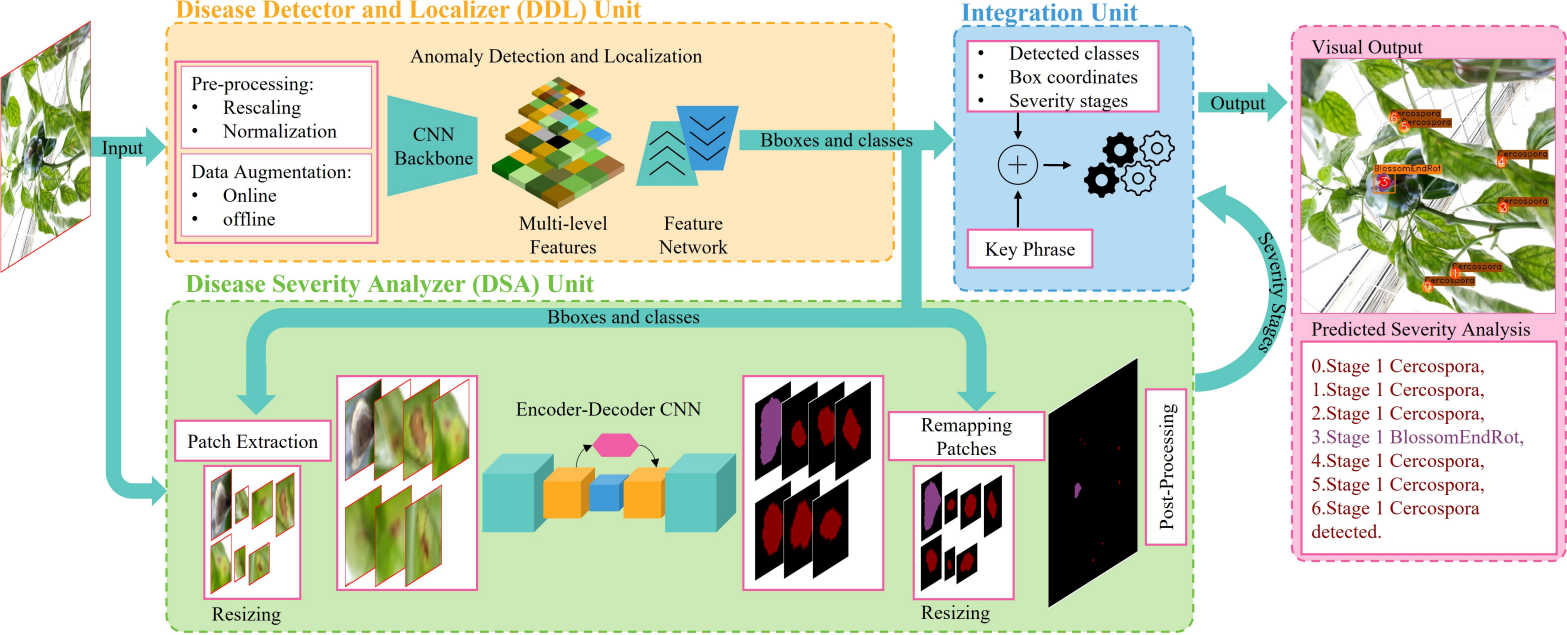
Flow diagram of the naïve framework. The overall architecture is composed of three main units. The DDL unit for generating reliable bounding boxes locating plant abnormalities. The DSA unit for analyzing and predicting intensity levels of abnormalities. An integration unit which combines the outcomes of both units to generate final detailed predictions.

We explore using the following design strategies to build a naïve framework that is just a patchwork of preexisting off-the-shelf object detection and segmentation networks in order to properly compare the naïve approach with our proposed approach. A flow diagram of the naïve framework is shown in **Figure 6**.

#### 4.2.1 Naïve DDL unit

Here, we consider a number of object recognition (OD) frameworks that can be tailored to fit with different combinations of CNN backbones (ResNet (He et al., 2016; Howard et al., 2017), MobileNet, etc.) and feature networks [FPN (Lin et al., 2017), PAN (Liu et al., 2018), BiFPN (Tan et al., 2020)] in order to detect anomalies and pinpoint where in the image they are located. Six SOTA OD pipelines were taken into consideration for trials based on their great performance and robustness. We experimented with numerous combinations of baseline feature extractors and different feature networks in each OD pipeline. It would enable conducting exhaustive evaluation experiments to contrast the effectiveness of the suggested methods with naïve ones.

#### 4.2.2 Naïve DSA unit

In the naïve framework, the purpose of the DSA unit is to recognize the intensity of all the diseases present in the plant by analyzing local regions provided by the DDL unit. We utilize a fully convolutional encoder–decoder network for this task, which performs a pixel-wise classification of the suspicious regions of plants and categorizes them according to their predicted disease severity stages. For this task, we utilize the architecture proposed in Ilyas et al. (2021) due to its superior performance on the plant disease recognition task and low memory footprint.

Further, we employ the same decoupled architecture of DDL and DSA units, as described in the previous section, for a performance comparison of both approaches (decoupled vs. coupled), to demonstrate how superior our proposed alternative is to the naïve approach, see *Results and discussion* for details.

Once the naïve DDL unit outputs a set of category-specific bounding boxes, we first extract the image regions pertaining to these bounding boxes. Then, we adapt the sizes of these regions to a fixed scale of 256 × 256, without preserving the aspect ratio. These rescaled regions are then passed through the naïve DSA unit to obtain fine segmentation maps. We then first resize these fixed-scale segmented regions back to their original resolution and remap them back onto the original image, as shown in **Figure 6**, before entering the integration unit.

Since there is no learning happening within the integration unit, the same integration unit is also employed for the naïve approach.

### 4.3 Experimental setup

**Figure 7:**
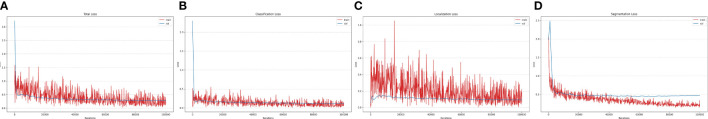
Loss curves of the DIANA framework. **(A)** Total loss, **(B)** classification loss, **(C)** localization loss, and **(D)** segmentation loss.

Experiments are conducted using our paprika plant dataset, which includes six annotated disease and pest categories, as well as 11 disease severity stages in total. Our dataset is partitioned into 70% training, 10% validation, and 20% testing sets to conduct the experiments. The training and validation sets are used for training and hyperparameter selection, respectively, while the testing set is used to evaluate the results on unseen data. The results reported here are an average of three independent runs; in each run, data are randomly split among train, validation, and test sets. All the experiments for the training and testing of our system are done on a Linux-based server with an intel Core i9-9940X 3.3-GHz processor having 3 NVIDIA RTX-2080 GPUs (for training) and 1 NVIDIA RTX-1080 GPU (for testing). The input resolution for all the models was selected based on available MS-COCO-pretrained weights. All the models were trained for 100K iterations. The detailed design specifications for our proposed disease analyzer (DIANA) are shown in **Table 3**. Training and validation losses of all output components in the DIANA framework are shown in **Figure 7**. All the curves show a similar trend, which means each component of the framework is learning its specific task jointly and smoothly.

**Table 3:** Specifications and hyperparameter settings for the DIANA framework.

	Value	Specification
DDL unit		
• M_bifpn_	3	–
• C_bifpn_	224	–
• M_box_	4	–
• M_class_	4	–
• N_a_	4	–
• N_cls_	6	–
• L_cls_	Focal loss	α = 0.25; γ = 1.8
• L_reg_	Smooth L1 loss	–
DSA unit		
• M_seg_	2	–
• N_ss_	11	–
• L_seg_	Focal Tversky loss	α = 0.3; β=0.7; γ = 4
Hyperparameters		
• Learning rate	0.08	Cosine decay, Warmup iterations 0-3K
• Optimizer	SGD	Momentum = 0.9
• Weight decay	L2	0.0005
• Dropout	Vanilla	0.3
• Batch size	3	–

### 4.4 Evaluation metrics

Our algorithm typically takes one image as input and outputs a set of disease regions along with severity levels. To effectively estimate anomaly categories and their location in the image, we employ three benchmark evaluation metrics to gauge the system’s performance. Both COCO-style and PASCAL-style mean average precisions (*mAPs*) are used as evaluation criteria for DDL tasks. Both classification accuracy and localization precision are measured by the *mAP*. We used a threshold of 50% to evaluate the intersection over union during localization.

In contrast, for the evaluation of the DSA task, we use two benchmark metrics mean intersection over union (*mIOU*) and mean panoptic quality (*mPQ*). Panoptic quality (Kirillov et al., 2019) can be seen as a combination of two different quality metrics called detection quality and segmentation quality. The panoptic quality is defined as


(7) $$PQ=\underset{Detection\;Quality\;\left(DQ\right)}{\underset{︸}{\frac{\left|TP\right|}{\left|TP|+\frac{1}{2}|FP|+\frac{1}{2}|FN\right|}}}\times \underset{Segmentation\;Quality\;\left(SQ\right)}{\underset{︸}{\frac{\sum_{(x,y)\in TP}IoU(x,y)}{\left|TP\right|}}}$$

Where, *x* represents the ground-truth segment and *y* represents the network’s prediction. From Equation 2, we can see that detection quality (DQ) is the F1 score which is actually the harmonic mean of precision and recall is used to evaluate instance detections, whereas segmentation quality (SQ) evaluates how close each detected instance is to its matched ground truth. Here, *IOU* measures how much the predicted segment (*y*) overlaps with the ground-truth segment (*x*). It is given by the following equation.


(8) $$IOU=\frac{x\cap^{}y}{x\cup^{}y}$$

## 5 Results and discussion

In this section, we assess the performance of the proposed approach for analyzing the severity of plant diseases. We arrange the experiments to support our statements.

### 5.1 Data augmentation

We leverage data augmentation to provide additional sample points in the dataset, lowering CNN generalization errors and boosting the network’s robustness toward unseen data. By preventing overfitting, data augmentation regulates the time period (and number of iterations) for which deep neural networks are trained. The summary of various data augmentation techniques is provided in **Table 4**. The results indicate that not every augmentation strategy improves performance. Some augmentation methods, such as changing the color temperature of the image and adding noise, have a negative impact on performance. Therefore, for subsequent experiments, we only utilize augmentation approaches that boost network performance.

**Table 4:** Effect of data augmentation on the performance of the DDL unit.

Augmentation	Parameters	mAP (%)
Baseline	–	75.38
**Geometric**		
• Random flip (left, right)	probability = 0.9	78.4
• Color temperature	t = [1,100, 10,000]	71.6
• Scale	x = [0.8, 1.2]; y = [0.8, 1.2]	79.83
• Translate	x = [0.2, 0.2]; y = [0.2, 0.2]	76.1
• Rotate	angle = [-30, 30]	77.4
**Distortion**		
• Median blur	k = [3, 7]	70.31
• Additive Gaussian noise	z = [0.0, 12.75]	74.69
• Additive Gaussian blur	sigma = [0, 1]	69.98
• Add to (hue, saturation, brightness)	a = [-8,15]	76.5
• Channel shuffle	probability = 0.55	78.76
**Advanced**		
• Cut and paste (Ghiasi et al., 2021)	obj_count = [5, 10]	81.97
• Mosaic (Bochkovskiy et al., 2020)	rows = [1,2]; columns = [1,2]	80.43

Faster-RCNN with ResNet-50 is used for all these experiments. Green color represents improvement from baseline, and blue color represents reduced performance from baseline.

### 5.2 Evaluation of the DDL task

We use the mean average precision (mAP) for the evaluation of all naïve DDL units (off-the-shelf ODs) and decoupled and coupled DDL units, i.e., DDL units decoupled/coupled with the DSA unit. The detailed results are reported in **Table 5**. As can be seen from **Table 5**, in the naïve framework, between two stage detectors, the Faster-RCNN architecture with the Inception-Res-Net backbone gives the best performance, whereas in the case of single-stage detectors, YOLOv5-x and Efficient-Det D3 performs on par with the Faster-RCNN’s top-performing variant. **Table 5** shows that the results achieved by our proposed framework, which was trained jointly for the DDL and DSA tasks, are superior to those of all other networks. Additionally, the same DDL unit when decoupled (decoupled DDL) and trained separately from the DSA unit showed performance equal to that of off-the-shelf ODs (naïve approach).

**Table 5:** DDL-unit performance on the Paprika Plant Disease Dataset (PASCAL VOC style AP).

Framework	Architecture	Backbone	Feature network	FLOPs	Params (M)	Speed (ms)	Batch size	Input resolution	Pascal style average precision						
									AP^BR^	AP^CP^	AP^GM^	AP^PM^	AP^SS^	AP^SM^	mAP^0.5^
Naïve framework	Faster-RCNN	ResNet-50	–	180B	42	65	16	1,024 × 1,024	0.8806	0.8583	**0.8697**	0.7436	0.7854	0.8249	0.8271
		ResNet-101	–	246B	60	72	8	1,024 × 1,024	0.8641	0.8250	0.8585	0.4055	0.5541	0.7972	0.7174
		Incep-ResNet	–	–	–	236	6	1,024 × 1,024	**0.9092**	0.8723	0.8640	0.7881	0.8465	0.8319	**0.8520**
	YOLOv4	CSPDarkNet53	PAN	119M	52.5	14	16	416 × 416	0.8919	0.8685	0.8677	0.7812	**0.8177**	0.8321	0.8432
		CSPDarkNet53-p7	PAN	–	280	28	16	512 × 512	0.8597	0.8526	0.8518	0.6984	0.8060	0.82	0.8147
	YOLOv5	CSPDarkNet53-s	PAN	16M	7.2	10	16	416 × 416	0.866	0.8460	0.85	0.7591	0.7744	0.8196	0.8192
		CSPDarkNet53-x	PAN	219M	88.4	20	16	512 × 512	0.8987	0.8913	0.8667	0.7817	0.8169	0.8336	0.8482
	SSD	MobileNet-V2	–			42	32	640 × 640	0.7924	0.8042	0.8168	0.6738	0.699	0.8181	0.7647
		MobileNet-V2	FPN			46	32	640 × 640	0.7957	0.7955	0.8203	0.7067	0.7005	0.8053	0.7707
	Retina-Net	ResNet-50	FPN	97B	34	87	16	1,024 × 1,024	0.8392	0.8495	0.8087	0.7522	0.8219	0.8364	0.818
		ResNet-101	FPN	127B	53	104	6	1,024 × 1,024	0.8366	0.8016	0.8024	0.6842	0.7611	0.8341	0.7867
	Center-Net	HourGlass-104	–	–	–	197	6	1,024 × 1,024	0.7836	0.7003	0.3496	0.5132	0.6404	0.6400	0.6045
	Efficient-Det	EfficientNet B0	BiFPN	2.5B	3.9	39	32	512 × 512	0.8741	0.8410	0.8227	0.7453	0.7828	0.8287	0.8158
		EfficientNet B1	BiFPN	6.1B	6.6	54	24	640 × 640	0.8954	0.8541	0.8445	0.7890	0.779	0.8412	0.8338
		EfficientNet B3	BiFPN	25B	12	95	8	896 × 896	0.9048	**0.8556**	0.8421	**0.7946**	0.8074	**0.8421**	0.8411
DIANA	Decoupled DDL	EfficientNet B3	BiFPN	25B	12	95	8	896 × 896	0.8978	0.8670	0.8538	0.7786	0.8166	0.8664	0.8467
	Coupled DDL	EfficientNet B3	BiFPN	17B	13.37	121	24	640 × 640	**0.9203**	**0.904**	**0.9351**	**0.9048**	**0.8786**	**0.9626**	**0.9176**

Results show the performance of both frameworks, i.e., naïve and DIANA. The feature network shows a framework using an additional CNN to combine multiscale features on top of the baseline backbone feature extractor, e.g., FPN or BiFPN. All models are trained and tested using the same protocol and on the same system. FLOPs show floating point operations required in billion (B) or in million (M); parameters are measured in million and speed measure in milliseconds (ms) provided only one instance of data is available. Batch size is decided on the basis of available resources (GPU memory). Input resolution shows the image’s input size and is decided based on the available pretrained weight’s (trained on COCO data). Green represents the highest score naïve framework, and blue represents the highest score achieved with DIANA.

**Figure 8:**
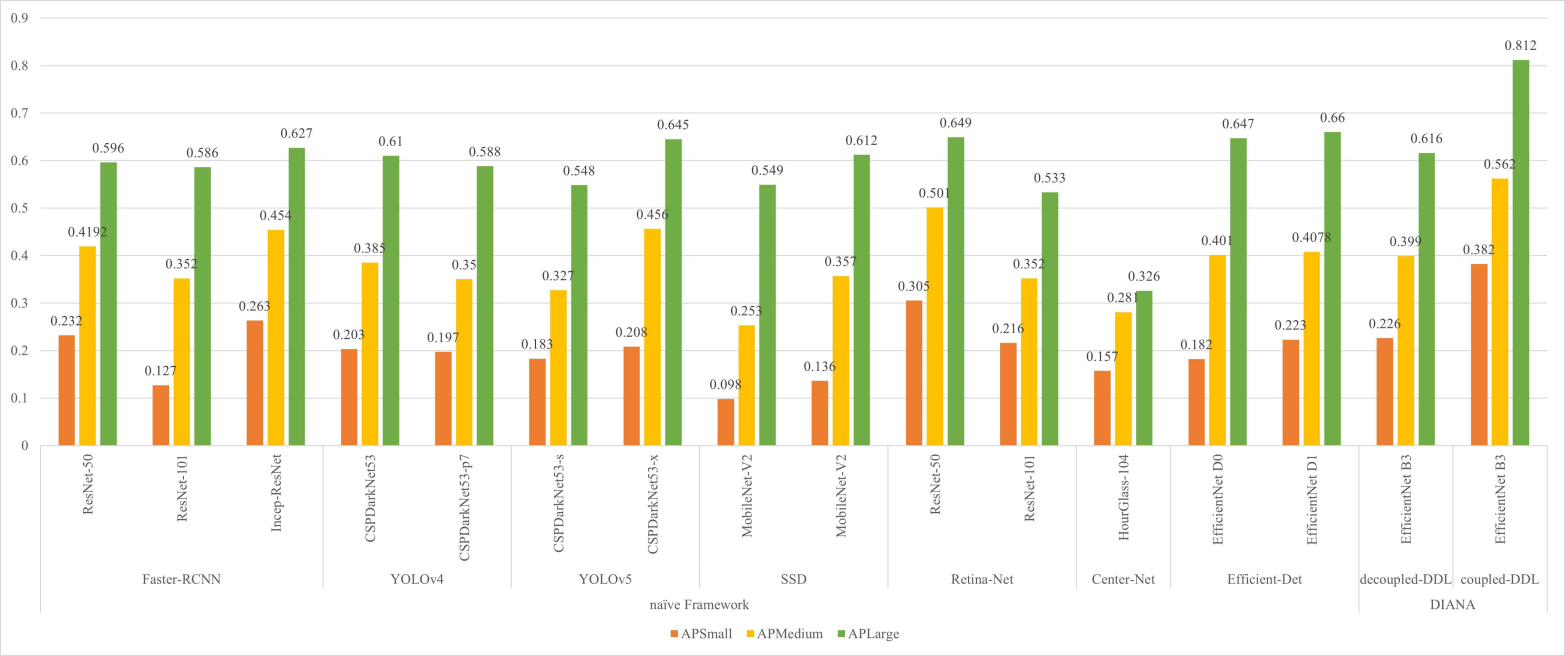
Effect of object size (anomaly region) on performance of proposed approaches.

In small object scenarios, performance analysis is crucial for various stages of diseases and pests having various sizes. Our proposed DIANA framework improves the detection rate of small objects by more than 10% as compared to the naïve approaches. In order to verify the detection effect of different-size anomaly regions, we define three different sizes of anomaly regions in our dataset. The anomaly sizes were divided into three subcategories: (i) small having area<32^2^ pixels, (ii) medium having area between [32^2^, 92^2^] pixels, and (iii) large anomalies with area >92^2^ pixels. We calculate the average precision (AP) of all different-sized objects, and the results are displayed in **Figure 8**. From **Figure 8**, it can be clearly seen that jointly training the DDL unit with the DSA unit greatly improves the detection rate of different-sized objects. As the DSA unit performs pixel-level classification on the varying-size feature maps along with incoming features from BiFPN, it also boosts the detection performance at levels of the feature pyramid.

**Figure 9:**
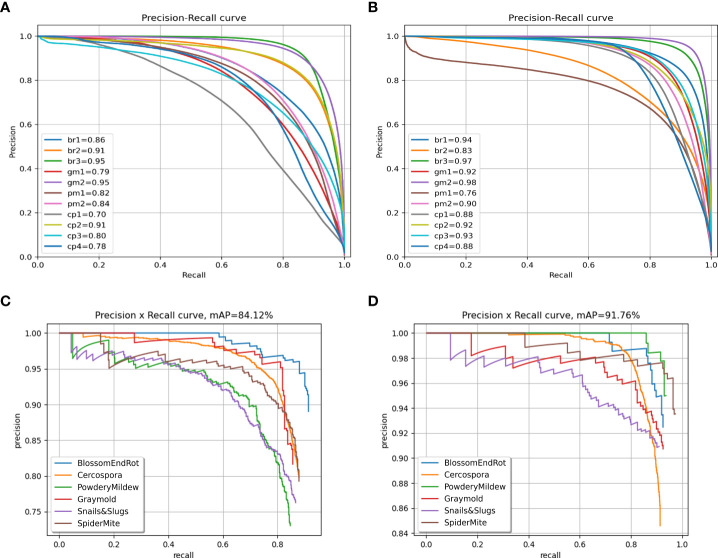
Precision-recall curves. **(A)** Decoupled DSA unit, **(B)** coupled DSA unit, **(C)** decoupled DDL unit, and **(D)** coupled DDL unit.

**Figure 10:**
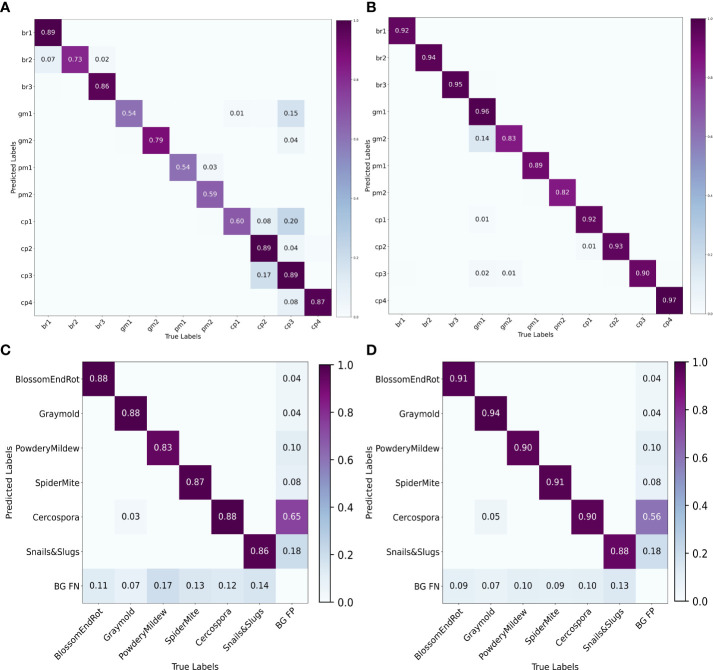
Confusion matrices. **(A)** Decoupled DSA unit, **(B)** coupled DSA unit, **(C)** decoupled DDL unit, and **(D)** coupled DDL unit.

We also plot the precision recall (PR) curve (**Figures 9** **C, D**) and confusion matrix (**Figures 10** **C, D**) of decoupled and coupled DDL units, to analyze the class-wise performance of the network. The PR curve also shows that the anomalies that have multiple instances inside a bounding box, e.g., powdery mildew, snail, and slugs, are most difficult to detect. As can also be seen in **Table 5**, simply increasing the CNN’s depth had a bad effect on the performance of the DDL task. Therefore, a framework that can extract reliable fine-grained multiscale features is needed rather than a network with increased depth. Furthermore, the confusion matrix demonstrates that training the DDL and DSA units jointly (coupled) decreases interclass confusion as well as total false positives and false negatives, especially in the case of the *Cercospora* class.

### 5.3 Evaluation of the DSA task

The detailed evaluation results for the DSA task on the paprika plant disease dataset are presented in **Table 6**. From **Table 6**, it is obvious that the DIANA frameworks outperform all the naïve-framework algorithms due to a global understanding of the task as a whole. The mean intersection over union (mIOU) metric only tells how much a predicted segment of a specific class overlaps with the ground-truth segment of that class. In contrast, the mPQ metric is the combination of both segmentation quality (showing overlap of segments) and detection quality (showing correct detection of classes), making it a stricter, more versatile, and reliable metric to evaluate the network’s performance. **Table 7** displays the per-class values of mPQ and mIOU for both decoupled and coupled DSA units. **Table 7** makes it evident that our system performs well for the DSA task on both evaluation metrics, with higher per-class performance.

**Table 6:** Evaluation of performance on the DSA task of both frameworks.

Framework	Model	mIOU (%)	mPQ (%)	FLOPs (B)	Param. (M)	Speed (ms)
Naïve framework	U-Net (Ronneberger et al., 2015)	68.7	45.4	60.5	4.65	31.2
	DeepLab_v2 (Chen et al., 2014)	71.05	47.3	24.03	3.09	20
	DeepLab_v3+ (Chen et al., 2017)	70.37	46.8	12.07	1.2	20.5
	DAM (Ilyas et al., 2021)	72.91	50.23	12.3	1.26	18.8
DIANA	decoupled-DSA	72.48	51.63	13.47	1.37	16.4
	coupled-DSA	87.7	70.78	–	–	121

We report class-wise and average (averaged over all classes) mIOU and mPQ.

**Table 7:** Class-wise performance of coupled and decoupled DSA units.

Architecture	Metric	br1	br2	br3	gm1	gm2	pm1	pm2	cp1	cp2	cp3	cp4	Mean
Decoupled DSA	mIOU	75.5	77.8	78.5	65.1	67.4	54.3	51.0	70.9	80.6	81.4	80.3	72.48
	mPQ	69.9	67.6	71.1	63.2	47.6	50.2	43.0	38.3	48.4	52.5	51.2	51.63
Coupled DSA	mIOU	87.0	91.2	92.9	91.2	88.7	79.7	80.1	83.9	92.6	89.6	88.6	87.77
	mPQ	81.8	79.7	83.5	84.5	67.8	71.3	69.0	41.8	65.8	68.3	65.1	70.78

To analyze the results in **Table 7** further, we plot the confusion matrix (**Figures 10** **A, B**) and precision recall curve (**Figures 9** **A, B**) for both decoupled and coupled DSA units on the DSA task. As can be seen from the confusion matrix in **Figure 10** **A**, even though the network is doing well in classifying the disease stages, there still exists some intra-class confusion, e.g., in the case of *Cercospora* (*cp*), the confusion is clearly visible between stage 1 (*cp1*) and stage 3 (*cp3*). We believe this is because there exists a huge intra-class variability in the *Cercospora* class, making it difficult to distinguish one severity stage form another. From **Figure 10** **B**, it can be seen that this intra-class confusion is significantly reduced while using the coupled DSA unit to generate severity predictions.

### 5.4 Ablation studies

It can be seen from **Figure 4** that while coupling the DDL and DSA units into one framework, feature maps from all pyramid levels of the final BiFPN layer are not passed onto the DSA unit. To investigate the impact of skip connections between a particular feature pyramid level (*P_l_*) and its corresponding encoder stage (*E_n_*), we carried out four independent experiments. The four possible configurations of such skip connections are shown in **Table 8**. The results are summarized in **Table 9**. The performance significantly increased from the baseline in the first experiment, by connecting all feature pyramid levels with all associated encoder stages utilizing the element-wise addition. The network performance then continued to improve when the coupling method from experiment 2 was used, but the computational load and memory footprint also considerably increased due to feature concatenation. We then followed the coupling strategies outlined in experiments 3 and 4 of **Table 8** and found that experiment 4 yielded best results with the smallest computational and memory footprint. Therefore, we adopted that strategy for coupling the DDL unit with the DSA unit.

**Table 8:** Possible ways of the coupling DDL unit with the DSA unit.

Exp. 1	Exp. 2	Exp. 3	Exp. 4
P_1_ →E_1_ P_2_ →E_2_ P_3_ + P_4_ →E_3_ P_5_ + P_6_→E_4_	P_1_ →E_1_ P_2_ →E_2_ P_3_^©^ P_4_ →E_3_ P_5_^©^ P_6_→E_4_	P_1_ →E_1_ P_2_ →E_2_ P_3_ →E_3_ P_5_ →E_4_	P_1_ →E_1_ P_2_ →E_2_ P_4_ →E_3_ P_6_→E_4_

Here, P_l_ represents the lth pyramid level, and E_n_ represents the nth encoder block.

**Table 9:** Ablation experiments for different coupling strategies.

Baseline	Exp.1	Exp.2	Exp.3	Exp.4	mIOU (%)	mPQ (%)
**✓**					72.48	51.63
**✓**	**✓**				79.34	67.18
**✓**		**✓**			85.98	69.74
**✓**			**✓**		77.6	65.39
**✓**				**✓**	**87.77**	**70.78**

### 5.5 Qualitative results

This section presents qualitative evidence of the proposed framework’s ability to produce accurate and reliable predictions given an image. We also show a few examples where the DIANA framework performs better than a naïve approach.

**Figure 11:**
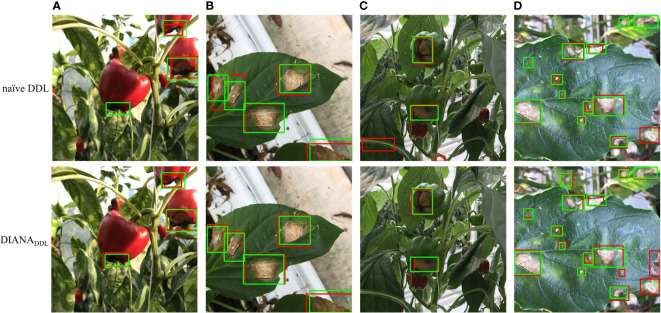
Comparison of qualitative results for coupled and decoupled DDL units.

First, we may infer from **Figure 11** the effects of joint optimization of the framework on both tasks. For instance, in **Figure 11** column (b), we can see that all the instances of Cercospora and gray mold disease are labeled correctly (green box), and the naïve DDL unit detected each instance correctly (red box). If we had not taken into account the disease severity levels, this would have been adequate. However, as stated earlier a single bounding box might contain diseases of varying degrees of severity. Therefore, a pixel-level subclassification of every detected region is necessary to prevent intra-subclass confusion. The left most bounding box contains two different stages of Cercospora disease (i.e., cp2 and cp3). The naïve DDL unit is unable to distinguish between two distinct subclass instances of the same class because of the hazy boundary between them. Whereas the DIANA framework due to its additional pixel-level subclassification branch successfully separates both instances, it also improves the performance of its coupled DDL unit. Moreover, as can be seen from column (a), (c), and (b) of **Figure 11**, the naïve DDL unit generates a lot of false positives and negatives than the coupled DDL unit.

**Figure 12:**
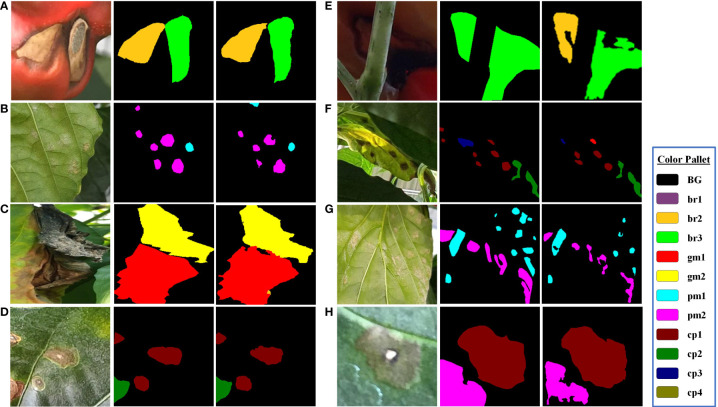
Visual results for disease severity analysis (DSA unit). The first column in each group displays input image patches, the second column displays ground-truth labels, and the third column shows predicted segmentation masks. The blue box at the side shows the color pallet used.

Some example of instances of the coupled DSA unit are shown in **Figure 12**. Rows (a, b, c, d) in **Figure 8** show that the coupled DSA unit can accurately detect and classify severity stages of different diseases present in the dataset. However, due to the complexity and high intra-subclass similarity of disease severity stages, the network sometimes fails to produce desired results. As shown in row (e) of **Figure 12**, in a single disease instance separated by a branch, one part is labeled as br3 (blossom end rot at stage 3) and another is labeled as br2. This kind of misclassification happens due to a reduced effective receptive field of network. The second issue is field-specific knowledge. Gray mold (gm) and Cercospora (cp) have similar visual and textural characteristics, as shown in **Figures 2**, **3**, but Cercospora can develop anywhere on the leaf, whereas gray mold only appears at the leaf’s edges. Because there is no optimal way to encode this type of information in a CNN, the network tends to confuse these two categories as evident by results in **Figures 10** **A** and row (f) of **Figure 12**. Moreover, in the case powdery mildew (pm), sometimes powdery colonies or yellow lesions that appear on the leaf’s surface do not have a clear boundary which makes it difficult for the network to generate well-defined segmented regions as shown in rows (g) and (h) of **Figure 12**.

**Figure 13:**
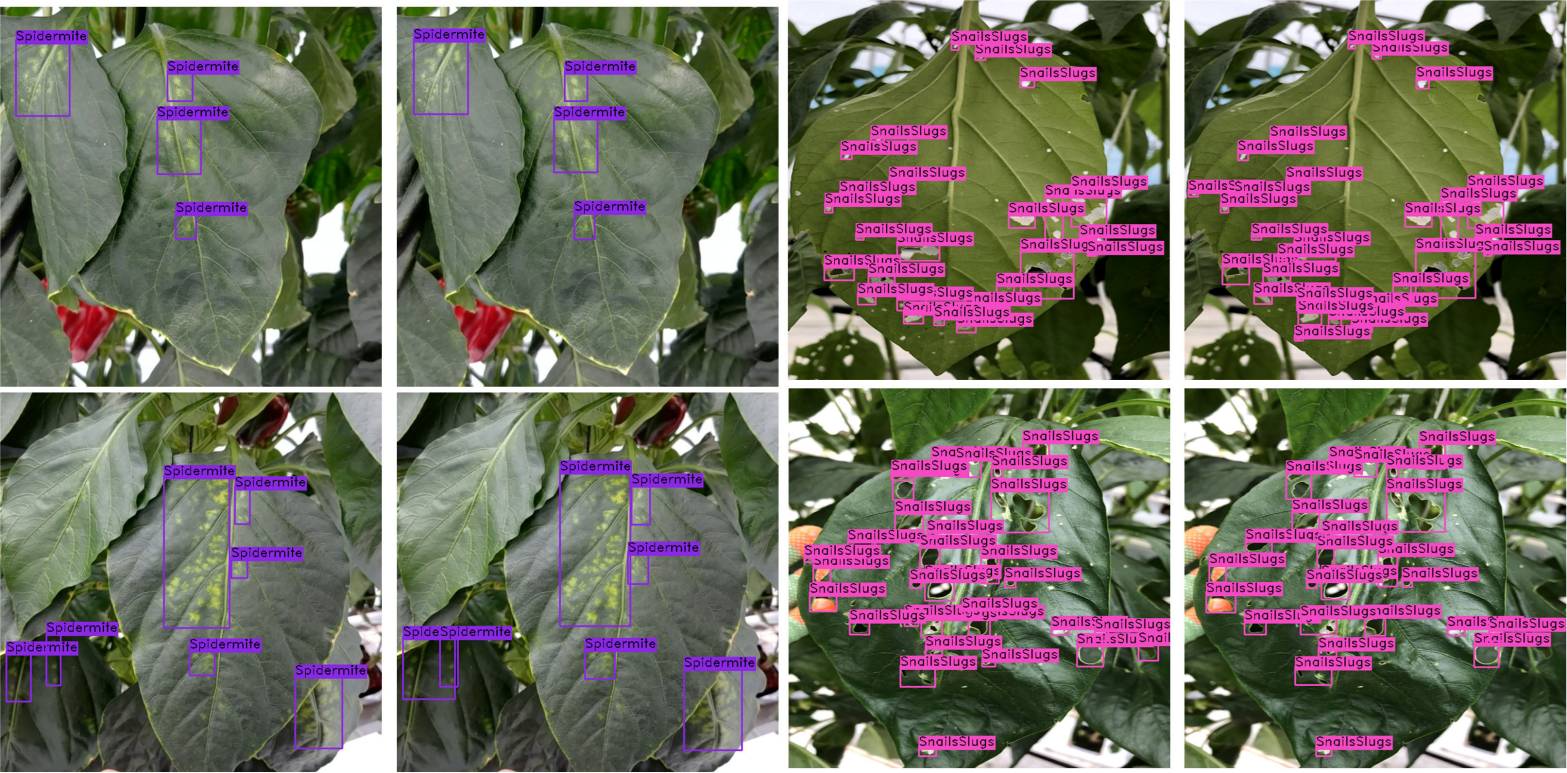
Visual results of DIANA in case only insect of pest damage is detected.

Next, we display the final output of DIANA; with an image given as input, it generates a set of bounding boxes that localize the anomalies, class types associated with the bounding boxes, and finally pixel-wise subclassified regions that display the severity stages of disease detected inside bounding boxes. For easier visualization and understanding of the results, we display the images in the following format. First is the input image with ground-truth labels overlaid over it; second is the network’s prediction with unique IDs assigned to all the detected disease instances by integration unit and finally a disease severity analysis window which displays key phrases describing the detected instances in the images with references to the assigned IDs. The color given to each phrase is related to the color pallet used for assigning colors to different disease severity stages as shown in **Figure 12**. **Figure 13** shows the visual output of DIANA, and **Figure 9** shows the output of the framework in case when only insect or pest damage was detected. More visual predictions are provided as supplementary material.

**Figure 14:**
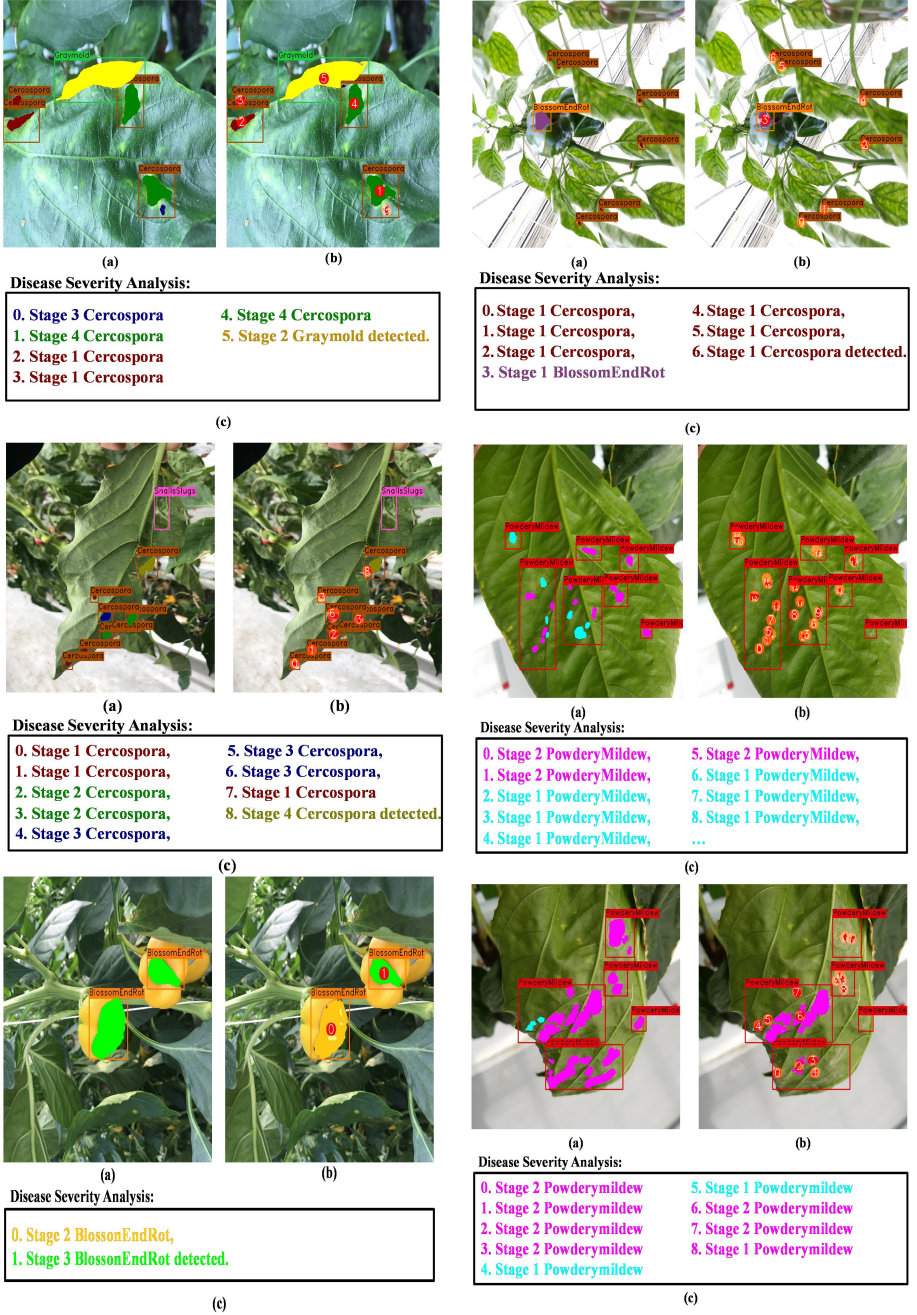
Visual results of DIANA on the paprika plant disease dataset. **(A)** Input image with ground-truth labels overlaid, **(B)** network output with unique ID assigned to each detected abnormal instance, and **(C)** severity analysis window displaying diagnosis results in user-friendly phrases. Each phrase states with an integer showing ID of the disease instance followed by the severity stage of the detected disease and finally the class of the detected disease.

**Figure 14** demonstrates that under a variety of real-world circumstances, our system can identify and categorize multiple diseases, as well as the phases of each disease’s severity. We also demonstrate certain instances in which our framework encounters issues when categorizing the stages of disease severity. For example, the last row of **Figure 14** shows that while the network was able to identify the disease category, it occasionally failed to identify powdery mildew instances because there are so many little, fluffy colonies with hazy borders. In cases when only insect–pest damage is found in the images, the inputs are not processed by the coupled DSA unit to save computational time; such examples are shown in **Figure 13** and the second row of **Figure 14**. As for the cases when both viral–fungal and insect–pest damages are detected on the plant, the coupled DSA unit is operated normally to get severity analysis, as shown in the second row of **Figure 14**.

### 5.6 Inference time vs. accuracy

**Figure 15:**
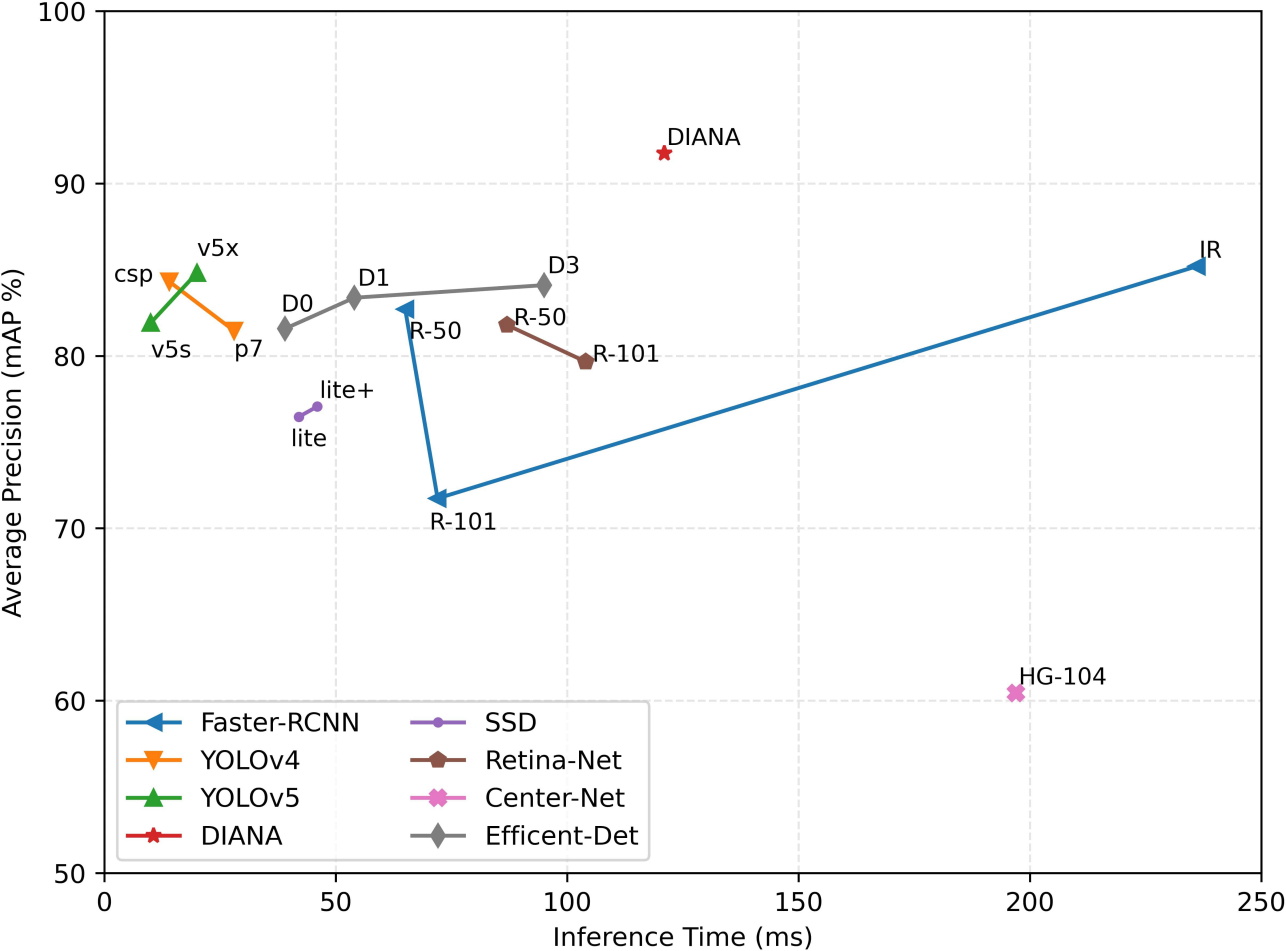
Inference time vs. model accuracy (mAP): all values are calculated using the same setup under the same conditions. Time is calculated in milliseconds (ms), and average precision is Pascal VOC style mAP at the 0.5 IoU threshold.

To conclude, we provide a summary of DIANA’s performance in comparison to other state-of-the-art object detectors in terms of inference time and accuracy achieved. For a fair comparison, the inference speed of all the ODs is measured under the same settings, i.e., on the same machine with an input batch size of 1. **Figure 15** shows the plot of inference time vs. model accuracy in terms of mean average precision. It can be seen clearly from **Figure 15** that our custom augmentation pipeline improves the network’s performance considerably. One thing worth mentioning here is that the specifications of both coupled units were considered when calculating the inference time, parameters, and FLOPs for DIANA.

## 6 Conclusion

We proposed an efficient disease severity analysis system in this study that can recognize and localize numerous plant diseases and pest damage, as well as provide the intensity of the diseases affecting the plant in the form of appropriate user-defined descriptive phrases. Our framework consists of three main units: (i) the first one being the DDL unit which accurately identifies and localizes all the anomalies affecting the plant, (ii) the second unit termed as the DSA unit which provides a reliable analysis of disease severities affecting the plant, and (iii) finally an integration unit which combines information from both these units and assigns unique IDs to all the detected anomaly instances along with generating the descriptive sentences, informing users about the current condition of the plant. We also discussed two possible approaches for combining these units into a single framework: one being a naïve approach in which neither of the three units communicate with each other, and another being the one in which two units are jointly trained and optimized in an end-to-end fashion for their respective tasks, resulting in a final disease analyzer (DIANA) framework. For this work, we also constructed a new dataset named paprika plant disease dataset, which includes three kinds of information, i.e., location, type, and severity of the abnormalities infecting the plant.

We conducted detailed ablation and evaluation experiments to verify and compare the performance of both approaches. We also compared the performance of two coupled units in DIANA separately with other top-performing models. For performance evaluation, we used three benchmark metrics, i.e., mAP, mIOU, and mPQ, and our network achieved 91.7%, 87.7%, and 70.78% scores, respectively. Moreover, in comparison to previous works, our system provides a more detailed and objective analysis of diseases infecting the plant. We introduced a reliable and cost-efficient tool that provides users (farmers) with technology that aids in crop management. We believe that this methodology will serve as a reference guide for future research in precision agriculture, as well as the construction of more effective monitoring systems to manage plant abnormalities, since the application can be easily extended to other field crops.

## Data availability statement

The raw data supporting the conclusions of this article will be made available by the authors at following link: https://github.com/Mr-TalhaIlyas/DIANA.

## Author contributions

TI conceived the idea, designed the algorithm, and wrote the manuscript. HJ contributed to data curation, formal analysis, and investigation. MS collected the original data and annotated it. HK, SL, and LC conceptualized the paper, supervised the project, and got funding. All authors discussed the results and contributed to the final manuscript.

## Funding

This work was supported in part by the National Research Foundation of Korea (NRF) funded by the Ministry of Education (NRF-2019R1A6A1A09031717 and NRF-2019R1A2C1011297) and the US Air Force Office of Scientific Research under Grant number FA9550-18-1-0016.

## Conflict of interest

The authors declare that the research was conducted in the absence of any commercial or financial relationships that could be construed as a potential conflict of interest.

## Publisher’s note

All claims expressed in this article are solely those of the authors and do not necessarily represent those of their affiliated organizations, or those of the publisher, the editors and the reviewers. Any product that may be evaluated in this article, or claim that may be made by its manufacturer, is not guaranteed or endorsed by the publisher.
